# Community differentiation of the cutaneous microbiota in psoriasis

**DOI:** 10.1186/2049-2618-1-31

**Published:** 2013-12-23

**Authors:** Alexander V Alekseyenko, Guillermo I Perez-Perez, Aieska De Souza, Bruce Strober, Zhan Gao, Monika Bihan, Kelvin Li, Barbara A Methé, Martin J Blaser

**Affiliations:** 1 Center for Health Informatics and Bioinformatics, New York University School of Medicine, 227 E 30th Street, 7th Floor, #740, New York, NY 10016, USA; 2 Department of Medicine, New York University School of Medicine, New York, NY 10016, USA; 3 Department of Microbiology, New York University School of Medicine, New York, NY 10016, USA; 4 Department of Dermatology, Harvard Medical School, Boston, MA 02114, USA; 5 Department of Dermatology, University of Connecticut, Farmington, CT 06032, USA; 6 J. Craig Venter Institute, Rockville, MD 20850, USA; 7 New York Harbor Veterans Affairs Medical Center, New York, NY, USA

**Keywords:** Cutaneous microbiota, Psoriasis markers, Microbiome analysis, Cutaneotypes

## Abstract

**Background:**

Psoriasis is a common chronic inflammatory disease of the skin. We sought to characterize and compare the cutaneous microbiota of psoriatic lesions (lesion group), unaffected contralateral skin from psoriatic patients (unaffected group), and similar skin loci in matched healthy controls (control group) in order to discern patterns that govern skin colonization and their relationship to clinical diagnosis.

**Results:**

Using high-throughput 16S rRNA gene sequencing, we assayed the cutaneous bacterial communities of 51 matched triplets and characterized these samples using community data analysis techniques. Intragroup Unifrac β diversity revealed increasing diversity from control to unaffected to lesion specimens. Likewise, principal coordinates analysis (PCoA) revealed separation of the lesion samples from unaffected and control along the first axis, suggesting that psoriasis is a major contributor to the observed diversity. The taxonomic richness and evenness decreased in both lesion and unaffected communities compared to control. These differences are explained by the combined increased abundance of the four major skin-associated genera (*Corynebacterium*, *Propionibacterium*, *Staphylococcus*, and *Streptococcus*), which present a potentially useful predictor for clinical skin type. Psoriasis samples also showed significant univariate decreases in relative abundances and strong classification performance of *Cupriavidus*, *Flavisolibacter*, *Methylobacterium*, and *Schlegelella* genera versus controls. The cutaneous microbiota separated into two distinct clusters, which we call cutaneotypes: (1) *Proteobacteria*-associated microbiota, and (2) *Firmicutes*-associated and *Actinobacteria*-associated microbiota. Cutaneotype 2 is enriched in lesion specimens compared to control (odds ratio 3.52 (95% CI 1.44 to 8.98), *P* <0.01).

**Conclusions:**

Our results indicate that psoriasis induces physiological changes both at the lesion site and at the systemic level, which select for specific differential microbiota among the assayed clinical skin types. These differences in microbial community structure in psoriasis patients are potentially of pathophysiologic and diagnostic significance.

## Background

Psoriasis is a chronic inflammatory skin disease of unknown etiology. The initial presentation and periodic exacerbations of psoriasis likely result from unidentified environmental exposures in individuals with genetic predisposition. The pathophysiology of psoriasis in part suggests an inappropriately activated cutaneous immune response directed against unascertained pathogens [1]. It is intriguing to surmise that in some patients, the colonizing microbiota of the skin elicit and perpetuate psoriasis. The identification of such ‘offending’ microbiota potentially could lead to early diagnostics, disease-modifying or, perhaps, curative therapies for this often devastating condition.

There have been only limited studies of the microbiota in psoriasis patients using molecular methods for the detection of bacterial and fungal taxa [2-5]. Such studies have involved relatively small numbers of subjects [2], relatively low-throughput bacterial community identification technologies [3-5] and unmatched study designs [2].

Study of the skin microbiota has been popularized by the availability of affordable high-throughput sequencing techniques for bacterial community identification. Variation in composition of the cutaneous microbiome has been studied from the ecological [6-8], anthropological [9], biomedical forensics [10], as well as medical standpoints [2,11-17]. Most notably, the major effort through the Human Microbiome Project (HMP) of the US National Institutes of Health (NIH) has resulted in microbial identification of communities in 300 healthy individuals across multiple body sites including several skin sites [16,18,19].

As one of the HMP demonstration projects, we sought to compare the cutaneous microbiota of psoriasis subjects with those from matched healthy controls in a disease-specific psoriasis cohort. The delineation of a psoriasis-specific microbiome signature is an attempt to understand a potential pathophysiologic influence of the microbiome on psoriatic disease. Further, if a specific microbiomal composition drives the psoriatic pathophysiology there would be potential to treat this disease by ‘normalizing’ the abnormal microbiome.

Because the cutaneous microbiota is complex, and its composition is site specific, we matched the affected lesion skin samples with unaffected contralateral skin samples from the same subject. For each psoriasis subject, a demographically matching control subject was selected and a specimen from that subject was obtained so as to match the affected body site. Thus, we collected and analyzed our data as triplets of lesion, clinically unaffected, and control specimens. To reduce the variability associated with treatment, we excluded subjects with recent antibiotic and other relevant treatments. A small subset of subjects was similarly followed longitudinally to study the effect of beginning antipsoriatic therapy on the composition of the microbiota.

## Methods

### Study population and subject specimen matching

Between June 2008 and September 2011, we obtained consent (using the model consent forms for the HMP demonstration projects) and enrolled a total of 199 subjects (75 patients with psoriasis and 124 healthy controls) with ethical approval from New York University School of Medicine Institutional Review Board (IRB #08-709). The subjects were recruited from the same geographic region (NYC) and same clinic at NYU. Among the patients with psoriasis, 57 (76%) had not been exposed to antibiotics or received treatment relevant to psoriasis for at least 1 month before skin samples were obtained. Among the healthy controls, 112 (90.3%) had not been exposed to antibiotics or received treatments relevant to psoriasis for at least 1 month before skin samples were obtained. Psoriasis subjects receiving antibiotics less than 1 month before enrollment were excluded from further analysis, only six (11.8%) of the remaining subjects had taken any antibiotics in the preceding year. When we reviewed the control subjects who actually were included, none had received antibiotics in the 12 months prior to sampling. A total of 54 (72%) patients with psoriasis were studied by swabbing of the affected (lesion) and unaffected (unaffected) sites (see section on Specimen collection for details).

For these subjects, we sought control subjects of the same gender and ethnicity, and of similar age (± 5 years), from whom a cutaneous specimen was obtained in a region proximate to the site of the psoriasis lesion. In total, we obtained matching specimens from 37 (29.8%) of the control subjects. One or more sites from each of these controls were matched to the lesions in the 54 subjects with psoriasis. A control subject could be matched to more than one patient, since we also matched for cutaneous site. However, each control cutaneous site was uniquely mapped to only one triplet, thus there was no duplication of specimens in the analysis. In each of 48 matched pairs, the 2 sites match, but in 6 sites we matched a back specimen with an abdomen, which are relatively similar in composition in healthy skin. The final analyses were performed on a set of 51 triplets, which had adequate depth of sequencing (>1,000 sequences per sample).

The resulting set of 51 triplets contained samples from sites that are characteristic of where typical psoriatic lesions occur in the general population. All of the sites were of the dry or sebaceous cutaneous microenvironments. We grouped the exact location of the specimen by proximity to other samples into four categories: body, head, upper extremities and lower extremities. Upper and lower extremities contained only samples of the dry cutaneous microenvironment, while all head samples and 8 out of 12 body trunk samples were characterized as sebaceous. A table describing the matching of psoriasis lesions to control sites and skin environment is provided in Additional file 1: Table S1.

Although psoriasis affects each gender equally, our final set of subjects consisted of 75% males. The bias towards men being sampled possibly can be explained by the fact that the medicines used in the study are often not used in women of childbearing age, thus limiting the enrollment of women.

#### Longitudinal study

A subset of psoriatic patients (n = 17) and age, gender, and ethnicity matched controls (n = 15) were followed prospectively for a period of 36 weeks and skin samples were obtained at baseline, and then after the cases started clinically indicated treatment for psoriasis, at 12 weeks, and 36 weeks (Additional file 1: Table S2). The 12-week mark was included in order to detect any initial effect of treatment on the microbiota, while the 36-week timepoint provided an ability to assess the stability of the changes observed at 12 weeks. For the 17 patients, the treatments were adalimumab (6), methotrexate (5), methotrexate and adalimumab (4), and other (2) (methotrexate and cyclosporine and adalimumab switched to Stelara (ustekinumab)). Although adalimumab blocks proinflammatory cytokines, whereas methotrexate alters adenosine metabolism, both agents have similar net effects in downregulating inflammation. As such, these two treatments may similarly affect the skin microbiota, through their shared anti-inflammatory effects, moving it to a more normal composition. While the goal of this study is to examine the maximal number of subjects with similar demographics, clinical skin condition, and treatment status, the potential differences in microbiota composition due to each treatment course may need to be studied in larger uniform cohorts.

### Psoriasis diagnosis and characteristics of populations

Patients were diagnosed with chronic plaque psoriasis in a dermatology clinic, and psoriasis was clinically classified based on characteristic morphologic features of the individual skin lesions and their distribution on the body. For each patient, disease duration, percentage cutaneous involvement, Psoriasis Area and Severity Index (PASI) and physician global assessment (PGA) scores were recorded. Means of severity scores for the subjects were PASI: 8.7 (± 10.1 SD), PGA: 6.6 (± 6.9 SD), and body surface area (BSA): 9.4 (± 13.9 SD). The characteristics of the control and affected study populations are given in Additional file 1: Table S3.

#### Specimen collection

In patients with psoriasis, we sampled a typical psoriatic plaque (designated as psoriasis, lesion), and as a reference site, a contralateral area of clinically uninvolved skin (designated as psoriasis, unaffected). We also examined skin from a healthy (control) person at the same approximate cutaneous location as the psoriatic lesion. We accomplished this by obtaining four skin swabs from each control person, from scalp (posterior-temporal, above ear crease), inner aspect of the elbow, lower lateral abdomen, and kneecap. This distribution mimicked the distribution of the lesions in most of the cases.

Methods for specimen processing have been described [20]. In brief, a 2 × 2 cm area of the cutaneous surface at each of the locations was sampled by swabbing the skin with a cotton pledget that had been soaked in sterile 0.15 M NaCl with 0.1% Tween 20 (Fisher Scientific, Fair Lawn, NJ, USA). DNA was extracted from the swab suspensions in a PCR-free clean room by using the DNeasy blood and tissue kit (Qiagen, Chatsworth, CA, USA); glass beads (0.5 to 1 mm) were added to the specimens and vortex mixed at maximum speed for 40 s, followed by DNA extraction, using the manufacturer’s protocol for genomic DNA isolation from Gram-positive bacteria, and samples were eluted in 100 μl AE buffer (DNeasy Blood and Tissue kit; Qiagen). To eliminate potential bacterial or DNA contamination of lysozyme, the lysozyme (Sigma-Aldrich, St Louis, MO, USA) was passed through a microcentrifuge filter (molecular mass threshold, 30,000 Da; Amicon, Bedford, MA, USA) at 18,514 *g* or 20 min before adding to the enzymatic lysis buffer.

### DNA sequencing and upstream processing

Samples were prepared for amplification and sequencing at the J. Craig Venter Institute (JCVI) Joint Technology Center (JTC) using a protocol for 16S rRNA gene amplification and sequencing developed as part of the NIH Human Microbiome Project [18,21]. Negative control experiments were performed, when we developed the extraction protocol with Qiagen kit [22]. In short, we used a reagent control that included all DNA extraction and polymerase chain reaction (PCR) reagents, including the sterile swab and the buffers, without the skin sample. This specimen was examined in parallel using the identical procedures as with the skin samples. After electrophoresis and ethidium bromide staining, preparations from these controls did not generate any visible bands. Negative control reactions were performed for every pool of amplicons to ensure no visible detection of amplicons on ethidium bromide stained agarose gels. The V1 to V3 region of the 16S rRNA gene was amplified using forward primer 5′-AGAGTTTGATCCTGGCTCAG-3′ attached to the Roche B adapter for 454-library construction and reverse primer 5′-CCGTCAATTCMTTTRAGT-3′ attached to the Roche A adapter and a 10-nt barcode (5′-A-adapter-N (10) + 16S primer-3′). The barcoded primer design was completed using a set of algorithms developed at the JCVI for these purposes [23,24]. PCR reactions were completed as follows (per reaction): 2 μl of gDNA (approximately 2 to 10 ng/μl), 1× final concentration of Accuprime PCR Buffer II (Invitrogen, Carlsbad, CA, USA), 200 nmol forward and reverse primers, 0.75 U of Accuprime TaqDNA polymerase high fidelity (Invitrogen), and nuclease-free water to bring the final volume to 20 μl. PCR cycling conditions consisted of an initial denaturation of 2 min at 95°C, 30 cycles of 20 s at 95°C, and 30 s at 56°C followed by 5 min at 72°C. A high number of amplification cycles is standard for skin studies because of typically low bacterial load in these specimens [25]. A negative control (water blank) reaction was examined after 35 cycles, and determined to be negative for the amplicon. Samples were then quantified, cleaned, and sequenced on the Roche 454-FLX (454 Life Sciences, Branford, CT, USA) as described previously [21], and a read processing pipeline consisting of a set of modular scripts designed at the JCVI were employed for upstream processing, consisting of deconvolution, trimming, and quality filtering, as described previously [26]. We performed a parallel analysis of the V3 to V5 16S rRNA gene region, but because of amplification and sequencing depth issues there were only 21 available triplets at this locus. Therefore, we focused exclusively on the V1 to V3 dataset.

### Downstream sequence processing and statistical analyses

After upstream processing and quality checking the passing sequences were analyzed using QIIME scripts [27]. We first clustered the sequences into 97% identity operational taxonomical units (OTUs) using the UCLUST program [28]. A representative sequence from each OTU cluster was used to assign taxonomy to the cluster using the RDP Classifier [29] executed at 80% bootstrap confidence cut-off. These representative sequences were further aligned using PyNAST [30] with the Greengenes core-set alignment template. We used the alignment to reconstruct an approximate phylogenetic tree using FASTTREE [31]. The obtained phylogenetic tree and abundance tables were used to calculate unweighted and weighted UniFrac β diversity indices [32]. The OTU absolute abundance table and UniFrac β diversity matrices were extracted from the pipeline for further analysis in the R statistical programming environment [33]. After processing the median sequencing depth per sample was 8,621 (IQR 5,013 to 11,412). The sequencing effort was statistically similar across clinical skin types, body sites and cutaneous microenvironment (Additional file 1: Figure S1).

Chimeras were checked with ChimeraSlayer [34]. In all, 2,700 of the total 34,123 OTUs (7.9%) identified in the study were marked as potentially chimeric. On average, the total relative abundance (fraction of total sequences) per sample of putatively chimeric sequences was 3% (± 2% SD). The abundance of suspected chimera was similar across clinical skin types, body sites and cutaneous microenvironment (*P* >0.05 Kruskal-Wallis analysis of variance (ANOVA)).

The rarefactions for richness and Shannon diversity indices were calculated using scripts based on the community ecology package vegan. Comparisons of intergroup and intragroup β diversity were performed using one-way ANOVA with the Tukey honestly significant difference (HSD) multiple comparison correction procedure.

We used the ade4 package [35] in R to perform Principal Coordinates Analysis (PCoA) on weighted Unifrac distances. To avoid negative eigenvalues in the analysis, we used the Cailliez method [36] to convert the weighted Unifrac distance matrix into a closest corresponding matrix with Euclidean properties, which was further used for PCoA.

Univariate testing was performed on OTU relative abundances, calculated by dividing the absolute abundances by the total sequence count per sample analyzed. Differential relative abundance of specific taxa and OTUs was calculated on highly abundant taxa (mean relative abundance >1%) using the Kruskal-Wallis test with FDR correction for multiple testing [37]. This approach is analogous to standard ANOVA in that the test is significant if any pair of relative abundances (control vs unaffected, control vs lesion, unaffected vs lesion) is different. *Post hoc* pairwise testing with additional multiple testing control can be utilized to determine which pair is different.

### Multiple testing correction and compositional data issues

The fact that the relative abundances present a compositional constraint violates the independence assumption. The relevant nature of the independence violation is that the individual significance values for the univariate tests are now potentially positively correlated. An example of such correlations is a situation where a statistically significant increase in one taxon abundance between two conditions is accompanied by a balancing decrease in one or more other taxa to have the abundances sum to a constant (1). However, the effect of this independence violation on the validity of the univariate findings is only mild for the following reasons: (1) the compositional constraint does not remove any true positive association, it only inflates the false negative rates, (2) false negatives are then controlled by the FDR multiple testing correction procedure, which is designed to take into account positive correlations in *P* values; (3) the effect of compositional constraint is minimized by the fact that we only focus on highly abundant taxa. Therefore, we believe that this study admits false positives at a rate similar to other genomic analyses, and this allows for discovery of useful associations with the phenotypes, which may be of potential diagnostic value, but may need further validation.

We utilized univariate χ^2^ tests to compare the prevalence of specific taxa among clinical skin types. Spearman correlation tests were used to find associations between severity scores and taxa abundance. The *P* values were adjusted for false discovery using the Benjamini-Hochberg procedure [37]. Receiver operating characteristic curves (ROC) were computed in R using the package ROCR [38] and significance of the classification signal as measured by the area under the ROC curve (AUC) was established by Mann–Whitney test.

To establish the presence and identity of cutaneotypes in our data, we utilized methodology identical to that previously used for gut-microbiota enterotype classification [39]. In short, we applied the partitioning around medoids (PAM) method [40] to the square root of the Jensen-Shannon divergence distances to compute optimal clustering with given numbers of clusters (2 through 20). The Calinski-Harabasz index [41] was used to establish the number of cutaneotypes to optimally cluster the data. Additional evidence for clustering was obtained using the gap statistic [42], and is described in supplementary materials.

Non-Euclidean multivariate analysis of variance (MANOVA) was used to analyze the association of microbiota with clinicodemographic variables [43]. This analysis utilizes a matrix of squares of arbitrarily computed pairwise distances in lieu of the covariance matrix to be decomposed into within and between group sums of squares. This decomposition is used to compute a pseudo-F-statistic, the significance of which may be established by permutation. *Post hoc* pairwise testing of significant multilevel factors was likewise performed by permutation. This analysis was performed using the adonis function from the R package vegan [44].

## Results

### Psoriatic lesions trend to decreased taxonomic diversity

**Figure 1:**
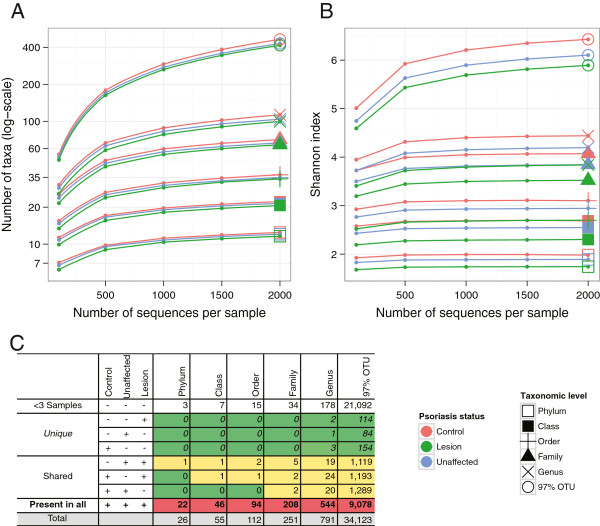
**α Diversity rarefaction curves of cutaneous microbiota in psoriasis (lesion), unaffected and control specimens. (A)** Taxonomical richness trends towards decreasing α diversity in unaffected and lesion specimens relative to control, with no statistically significant differences between skin types. **(B)** Shannon index is significantly different (decreases from control to unaffected to lesion) among skin types at all taxonomic levels (*P* <0.05), except at the operational taxonomical unit (OTU) level. **(C)** Analysis of taxa sharing. Taxa present in <3 samples excluded from the analysis. Taxa that are only observed in one clinical skin type are denoted as ‘unique’. Taxa that are present in two types of skin are denoted as ‘shared’. The data show that nearly all taxa are represented in all three types of skin. The shading represents the relative distribution (heatmap) for each column number (green = low, yellow = intermediate, red = high).

To assess the changes in α diversity related to psoriasis, we examined the diversity of cutaneous microbiota at the three clinical skin types, in terms of taxonomical richness and evenness at phylum, class, order, family and genus taxonomical level, as well as at the level of OTUs defined as sequences with 97% identity to each other. At all taxonomical levels, the richness index demonstrated a trend of decreasing diversity from controls to unaffected to psoriatic lesion skin (Figure 1A). We tested the significance of this trend at depth of 2,000 sequences per sample; however, this trend was not statistically significant (*P* >0.05). The shape of saturation curves suggests that while our data accurately reflect genus-level and higher-level composition of cutaneous microbiota, our data do not encompass all subgenera-level skin taxa. In terms of evenness as measured by the Shannon index (also known as non-normalized Shannon equitability index) (Figure 1B), saturation was reached faster at each taxonomic level. Lesion specimens had less evenness (as measured by the Shannon index) than the unaffected and control specimens; we observed statistically significant differences in evenness (*P* <0.05 at the phylum, class, order, family and genus levels, however the differences were not significant at the 97% identity OTU level, *P* >0.05). The specimens from all three clinical skin types share a large fraction of taxa at all taxonomical levels (Figure 1C). Essentially no taxa uniquely characterize any of these skin types. Decreased evenness in the lesion microbiota along with maintenance of approximately the same number of total taxa predict that one or more taxa should exhibit higher relative abundance in the lesion samples, compared to the other groups.

### Psoriasis status is associated with relative abundance and presence of specific taxa

**Figure 2:**
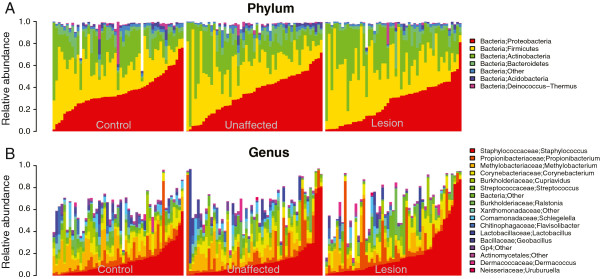
**Taxonomical composition of cutaneous microbiota shown by skyline plots. (A)** phylum, **(B)** genus levels. Predominant taxa are shown. White areas reflect specific taxa not graphed.

Next, we examined the relative abundance of the taxa that constitute the multivariate distribution of the cutaneous microbiota. Skyline plots demonstrated generally similar compositions of cutaneous microbiota in terms of phylum-level relative abundance (Figure 2A) for the three groups of specimens. We observed that three phyla: *Proteobacteria*, *Firmicutes*, and *Actinobacteria* dominate the skin microbial communities in all three skin types, consistent with observations of previous studies of psoriasis and healthy skin composition [2]. At the genus level (Figure 2B), lesional specimens show similar composition to other groups with no strongly apparent differences. We further examined the dominant taxa (those with mean relative abundance of ≥1%) at each taxonomic level to assess their association with psoriasis status (Table 1 and Additional file 1: Figure S2). Many taxa were found to be associated with psoriasis status, despite high variance in each clinical group. Surprisingly, all of the highly abundant taxa that were significantly different showed a decrease in relative abundance in lesion specimens.

**Table 1:** Univariate association of major taxa with psoriasis status using Kruskal-Wallis ANOVA

**Taxonomic level**	**Taxon**	**Kruskal-Wallis χ**^**2**^	**df**	**Median abundance**			**FDR adjusted *P *** **value**^**a**^
				**Control**	**Unaffected**	**Lesion**	
Phylum	Bacteria;Acidobacteria	18.68	2	0.97%*	0.48%**	0.18%***	6.14E-04
	Bacteria;Deinococcus-Thermus	9.14	2	0.37%*	0.22%**	0.14%***	2.41E-02
	Bacteria;Other^b^	14.27	2	2.52%*	2.20%**	1.42%***	2.79E-03
	Bacteria;Proteobacteria	7.42	2	33.32%**	36.21%*	30.75%***	4.27E-02
Class	Acidobacteria;Acidobacteria_Gp4	14.08	2	0.92%*	0.41%**	0.12%***	4.39E-03
	Bacteroidetes;Sphingobacteria	8.53	2	1.70%*	1.50%**	0.74%***	3.52E-02
	Deinococcus-Thermus;Deinococci	9.14	2	0.37%*	0.22%**	0.14%***	3.44E-02
	Proteobacteria;Alphaproteobacteria	7.63	2	16.44%*	11.37%**	7.64%***	4.41E-02
Order	Acidobacteria_Gp4;Gp4	14.08	2	0.92%*	0.41%**	0.12%***	5.70E-03
	Sphingobacteria;Sphingobacteriales	8.53	2	1.70%*	1.50%**	0.74%***	3.05E-02
	Alphaproteobacteria;Rhizobiales	11.53	2	13.63%*	7.94%**	4.11%***	1.36E-02
	Betaproteobacteria;Burkholderiales	8.80	2	9.33%**	11.61%*	5.95%***	3.05E-02
	Gammaproteobacteria;Xanthomonadales	9.34	2	1.83%*	1.23%**	0.32%***	3.05E-02
Family	Gp4;Other	14.08	2	0.92%*	0.41%**	0.12%***	9.22E-03
	Sphingobacteriales;Chitinophagaceae	10.61	2	0.93%*	0.56%**	0.34%***	1.74E-02
	Bacillales;Bacillaceae	11.75	2	0.61%*	0.31%**	0.09%***	1.18E-02
	Rhizobiales;Bradyrhizobiaceae	12.95	2	2.21%*	1.89%**	0.73%***	1.08E-02
	Rhizobiales;Methylobacteriaceae	12.07	2	8.43%*	4.42%**	2.39%***	1.18E-02
	Burkholderiales;Burkholderiaceae	8.46	2	4.89%*	4.68%**	1.95%***	3.82E-02
	Burkholderiales;Comamonadaceae	7.71	2	2.05%*	1.64%**	0.94%***	4.94E-02
	Xanthomonadales;Xanthomonadaceae	9.61	2	1.79%*	1.23%**	0.31%***	2.45E-02
Genus	Gp4;Other	14.08	2	0.92%*	0.41%**	0.12%***	2.98E-03
	Chitinophagaceae;Flavisolibacter	14.32	2	0.22%*	0.05%**	0.02%***	2.98E-03
	Bacillaceae;Geobacillus	8.03	2	0.06%*	0.00%***	0.00%***	3.83E-02
	Methylobacteriaceae;Methylobacterium	12.19	2	8.43%*	4.42%**	2.39%***	5.48E-03
	Burkholderiaceae;Cupriavidus	21.50	2	3.20%**	3.38%*	0.73%***	1.83E-04
	Comamonadaceae;Schlegelella	25.21	2	0.64%*	0.35%**	0.05%***	5.71E-05
	Xanthomonadaceae;Other	12.74	2	1.46%*	0.39%**	0.17%***	4.85E-03
Operational taxonomical unit (OTU)	Methylobacterium;484	15.16	2	5.51%*	2.65%**	1.22%***	1.66E-03
	Gp4;3855	16.95	2	0.72%*	0.06%**	0.00%***	9.04E-04
	Xanthomonadaceae;6162	10.04	2	1.06%*	0.16%**	0.02%***	1.72E-02
	Cupriavidus;6869	20.96	2	2.38%**	2.40%*	0.55%***	1.83E-04
	Schlegelella;13613	26.57	2	0.47%*	0.22%**	0.00%***	2.21E-05
	Methylobacterium;24283	9.11	2	1.02%*	0.74%**	0.34%***	2.28E-02

Coding indicates high (*), intermediate (**), or low (***) abundance values in each row.

^a^Only taxa significant at 5% FDR are shown.

^b^This taxon represents all sequences that were not assigned to a known phylum. It is significant at all taxonomic levels.

We examined the abundances of the major skin genera with respect to psoriasis status. Each of the major taxa that typically are found on skin (*Propionibacterium*, *Corynebacterium*, *Streptococcus*, and *Staphylococcus*), were not significantly different between lesion, unaffected, and control. However, the combined relative abundance of these four genera was significantly (*P* <0.01) different across the specimen groups. Upon further examination *Propionibacterium* does not play an important role for distinguishing skin types. Combined relative abundance of just three genera (*Corynebacterium*, *Streptococcus*, and *Staphylococcus*) attained statistical significance (*P* <0.05). The mean combined relative abundance of these genera increases from control (mean (± SEM): 22.03% (± 2.1%)) to unaffected (22.9% (± 2.5%)) to lesion (33.8% (± 3.3%)) specimens. Pairwise *post hoc* testing revealed that the combined abundance of the three genera in control and unaffected microbiota was different from lesion (*P* <0.05). Likewise, the univariate classification signal, as measured by AUC, for each of the four skin-associated taxa was not significant, while the combined signal of all four and just three (without *Propionibacterium*) as well as the univariate signals of other named differentially abundant taxa were stronger, approaching diagnostically relevant values (AUC 0.65 to 0.81) (Additional file 1: Figure S3). To place these results in context, the widely used prostate-specific antigen (PSA) has an AUC <0.75, and for some versions <0.65 [45]. We have further analyzed this and an additional dataset for multivariate signatures of psoriasis and the results suggest that even stronger reproducible signals are possible [46].

We utilized univariate χ^2^ to identify individual OTUs of potential diagnostic significance. These tests for presence/absence of highly abundant OTUs (mean relative abundance ≥1%) showed that after correction for false discovery rate (FDR), 2 specific OTUs (out of a total of 13 highly abundant OTUs) were strongly associated with psoriasis status (*Acidobacteria Gp4* (OTU 3,855, *P* <0.002) and *Schlegelella* (OTU 13,613, *P* <0.00002). The identified OTUs were frequently encountered in all skin types (Additional file 1: Table S4), despite the evidence for differential prevalence according to specimen type. The combined double-positive psoriasis predictor based on these two OTUs (representative sequences shown in Additional file 1: Table S5) was associated with psoriasis status (*P* <10^-7^) and achieved a diagnostically relevant odds ratio (0.073) (95% CI 0.024 to 0.203) for distinguishing lesion versus control samples (Table 2). From these data, we conclude that it is feasible to identify strong predictive markers of psoriasis from composition of cutaneous microbiota.

**Table 2:** Diagnostic performance of incidence-based psoriasis predictor

**Group**	**Positive for OTU (%)**^**a**^		**Double positive**^**b**^	**Odds ratio**	
	***Acidobacteria Gp4 *** **(3,855)**	***Schlegelella *** **(13,613)**		**Lesion (95% CI)**	**Unaffected (95% CI)**
Control	80	96	78	0.073 (0.024 to 0.203)	0.329 (0.123 to 0.838)
Unaffected	61	69	53	0.220 (0.080 to 0.566)	
Lesion	39	49	20		

^a^The only two taxa significant at 5% FDR within all OTUs with >1% mean abundance.

^b^χ^2^ = 33.9, df = 2, *P* value <0.0000001.

*OTU* operational taxonomical unit.

### Psoriasis lesions are characterized by greater intragroup variability

**Figure 3:**
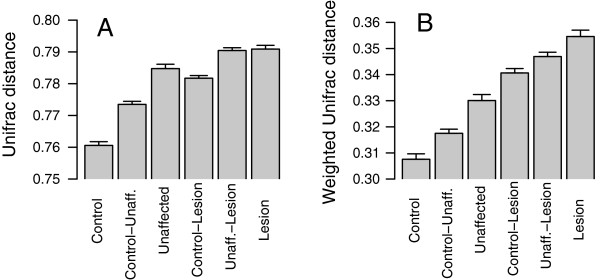
**Intergroup and intragroup β diversity.** Mean (± SEM) pairwise Unifrac distances obtained by unweighted **(A)** or weighted **(B)** methods are shown. Significance was determined by one-way analysis of variance (ANOVA) with the Tukey honestly significant difference (HSD) method for correction for multiple comparisons. For the 51 triplet sets, all differences in the unweighted Unifrac comparisons were significant (*P* <0.05), except for the comparisons between control-lesion and within unaffected, and the comparison between unaffected-lesion and within lesion. All differences between the weighted pairwise comparisons were significant (*P* <0.05) except for the comparisons between control-lesion and unaffected-lesion, and the comparison between unaffected-lesion and within lesion.

We evaluated the skin microbiota for the degree of sample-to-sample variability using β diversity, a distance-based ecological measure that allows for comparison of specimens grouped according to skin type. The β diversity analyses were performed, using subject site as the unit of independent analysis, however, for lack of appropriate comparison methodology these analyses do not account for the fact that some control subjects contributed multiple specimens (from different sites) to the matched triplets. We expect the intrasite differences to be greater than the differences between subjects sampled at a single site, which at least partially alleviates this apparent loss of independence. We chose to examine the β diversity in our data using weighted and unweighted Unifrac dissimilarity measures [32] based on their performance [47] in showing the differences among clinical groups. In terms of both measures, lesional specimens showed the highest intragroup diversity, followed by the unaffected, and control specimens (Figure 3). These findings indicate that control subjects are more similar to one another in terms of their cutaneous microbiome than the specimens from unaffected sites of psoriasis patients, and that each lesion harbors a more distinct community of microbes compared to other lesions. The intergroup unweighted Unifrac distances are smaller between lesion and control than between lesion and unaffected sites. Similarly, the unaffected skin is closer to the control than to lesion. These findings suggest that the microbiota of unaffected skin from psoriasis patients maintains many compositional characteristics of the skin of the control subjects, while simultaneously showing divergence from lesion skin on the contralateral part of the body. This difference is indicated by the substantial β diversity of the lesional microbiota. Using the weighted Unifrac analyses, trends are similar, with increasing β diversity for lesion > unaffected > control specimens (Figure 3B). Distances in relation to the lesion specimens are greater in relation to the control and unaffected specimens than are the comparisons of control and unaffected. From these findings, we conclude that psoriasis is associated with a systemic change in the cutaneous microbiota that is evident in both the lesion samples and, to a lesser degree, in the clinically unaffected skin.

### The psoriatic microbiota is associated with a cutaneotype enriched for Firmicutes and Actinobacteria

**Figure 4:**
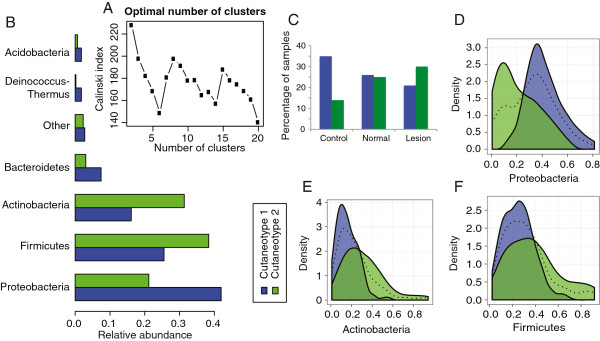
**Evidence for psoriasis-associated cutaneotypes. (A)** Clustering analysis of the Jensen-Shannon divergence distances indicated that two clusters, which we called cutaneotypes, can optimally describe the data. The Calinski-Harabasz index plot versus the number of clusters was used to select the optimal number of clusters, which also indicated that two has the highest Calinski index. **(B)** The relative abundance of major phyla is different between the two cutaneotypes. **(C)** The distribution of specimens in the three clinical categories is significantly associated with cutaneotype (*P* <0.01 based on χ^2^ test). Cutaneotype 2 is enriched for lesion specimens, while controls are more likely to be of cutaneotype 1 (OR 3.52, 95% CI 1.44 to 8.98), unaffected skin specimens are approximately evenly distributed between the cutaneotypes. Cutaneotype 1 is dominated by *Proteobacteria***(D)**. In the density plots, the dotted line shows the combined density for both cutaneotypes: blue, cutaneotype 1; green, cutaneotype 2. In cutaneotype 2, *Actinobacteria***(E)** and *Firmicutes***(F)** are the dominant phyla.

For clinical and investigational purposes, it would be useful if the cutaneous microbiota could be stratified into distinct subtypes. We performed a preliminary analysis of the cutaneous microbial community data for evidence that there might be such stratification into distinct clusters, using the methodology previously employed to report clustering of the gut microbiota [39]. Based on the Calinski-Harabasz index [41], we show that the data can be optimally separated into two clusters, which we call cutaneotypes (Figure 4A). A representation of these clusters on the first PCoA plane is shown in Additional file 1: Figure S4. These cutaneotypes differ in terms of the relative abundance of major phyla (Figure 4B); specifically, cutaneotype 1 is dominated by *Proteobacteria* (Figure 4D), while communities of cutaneotype 2 have higher relative abundance of *Actinobacteria* (Figure 4E), and *Firmicutes* (Figure 4F). We notice that the distribution of *Proteobacteria* is bimodal, with peaks corresponding to the modes of the respective contributing distributions, each linked to a cutaneotype. This suggests that changes in *Proteobacteria* may be driving the clustering, while the changes in relative abundance of the other two phyla may be due to compositional effects. Although the actual value of *Proteobacteria* relative abundance may vary continuously over the domain (0% to 100%), the bimodality of the *Proteobacteria* distribution serves as an important additional piece of evidence for the presence of two distinct clusters. The peaks of the modes correspond to the modes of the underlying distributions. However, the exact delineation between cutaneotypes may be difficult to define because the two distributions contributing to the bimodal mixture have substantial overlap in their tails.

In addition to differing in terms of taxonomic composition, the cutaneotypes are associated with psoriasis status (*P* <0.01). Non-lesion specimens from affected individuals have approximately even assignment to cutaneotypes, while most of the control specimens belong to cutaneotype 1 and the lesional specimens are dominated by cutaneotype 2 (Figure 4C). The difference in cutaneotype composition between the lesion and control samples yields a high odds ratio (3.52 (95% CI 1.44 to 8.98)). The evidence for existence of these two distinct cutaneotypes in the data is further corroborated by analyses based on the gap statistic [42] (Additional file 1: Figure S5 and Supplemental Methods section). In total, these findings provide strong support of the existence of distinct types of skin microbiota, cutaneotypes that differ in prevalence in psoriasis. Cutaneotypes serve as the description of the internal structure of our dataset, which may or may not be reproduced in other data. We caution the reader against overgeneralization of the reported preliminary observations to other datasets. The utility of clustering analysis rests in part on its reproducibility and generalizability; future studies of the cutaneous microbiota should examine this issue.

### Psoriasis status is the major source of variability in microbial communities

**Figure 5:**
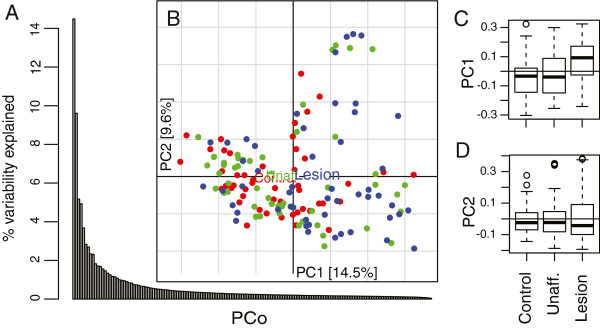
**Principal coordinates analysis (PCoA) of weighted Unifrac distances.** Based on separation of the relative values of the percentage variability explained by the principal axes, we chose to represent the cutaneous microbiome of our study in the first two components **(A)**. **(B)** On the scatterplot of the first two principal axes of the PCoA, each point represents an individual subject (red = control, green = unaffected, blue = lesion) and the colored boxes are positioned at the geometrical center of all the points in the corresponding groups. The distributions of the points along PC1 **(C)** and PC2 **(D)** are shown in the boxes (representing the middle 50% of the data) and whiskers (95% interval) plots. Along PC1, lesion samples are significantly different from control (*P* <0.001) and unaffected (*P* <0.015) based on one-way analysis of variance (ANOVA) with Tukey honestly significant difference (HSD) multiple comparison correction.

We next attempted to decompose the variability of the microbiota into major components to identify possible associations with psoriasis status. We used PCoA on weighted Unifrac distances to examine the association of the entire cutaneous microbiome with specimen type (Figure 5). The first two axes are clearly separated from the rest of the components (Figure 5A), therefore we chose to represent the cutaneous microbiome of our study in these two dimensions. Only approximately 24% of total variation in the dataset is explained by the first plane, indicating that most of the variation is captured in multiple other dimensions. The high variability of the composition of individual cutaneous microbiota is indicated by the observation that 11 and 84 axes are necessary to represent, >50% and >90%, respectively, of the variability present in the specimen comparisons (data not shown). We tested the first two principal coordinates for association with psoriasis status. The microbiota from the lesions is distinguishable from that of unaffected and control skin along the first principal axis (Figure 5B) (*P* <0.001, ANOVA). This means that the major axis of variability as determined by Unifrac-based principal coordinates analysis is aligned with psoriasis status. Samples from control and unaffected skin are located across the origin from the lesion samples on PC1, but not on PC2 (Figure 5C). The individual samples from the different specimen types occupy common areas on two-dimensional and three-dimensional plots, without clear clustering according to psoriasis status. These findings reiterate the increased diversity in the psoriasis lesions and establish psoriasis status as one of the main sources of variability in our datasets, despite the substantial remaining variation unaccounted for by the clinical data.

We next analyzed the cutaneous microbiota for association with clinicodemographic variables, to identify possible interactions with psoriasis status using non-parametric multivariate analysis of variance (adonis) (Table 3). The model for explaining the observed variability of the microbial communities (β diversity) included the following explanatory variables: psoriasis status, cutaneotype, month and year of sampling, body site, skin microenvironment type, age, gender, ethnicity of the subjects, and family history of psoriasis. Interactions of the above variables with psoriasis status were also tested.

**Table 3:** Non-Euclidean multivariate analysis of variance

**Factor**	**df**	**Sum of squares**	**Pseudo-F-statistic**	***P *** **value**^**a**^
Body site^b^	3	1.363	1.624	**0.00015**
Cutaneotype	1	0.626	2.236	**0.00015**
SampleMonthYear	24	10.141	1.510	**0.00015**
Status	2	0.854	1.526	**0.0003**
Skin micro-environment	1	0.321	1.141	0.11000
Gender	1	0.363	1.296	0.2940
Ethnicity	3	0.972	1.158	0.3179
Age	1	0.305	1.091	1.0
History	1	0.302	1.080	1.0
Status × body site	6	1.529	0.910	1.0
Status × age	2	0.482	0.862	1.0
Status × sample month year	28	7.265	0.927	1.0
Status × gender	2	0.468	0.837	1.0
Status × ethnicity	6	1.733	1.032	1.0
Status × history	2	0.490	0.876	1.0
Status × cutaneotype	2	0.549	0.981	1.0
Residuals	66	18.471		
Total	150	45.913		

^a^*P* values are based on 100,000 permutations and are adjusted for multiple testing using the Bonferroni method. Significant values are in bold.

^b^Body sites are grouped into head, body, upper extremities, lower extremities.

We did not find evidence that links subject gender, ethnicity, age, or family history of psoriasis with the variability of the cutaneous microbiota. However, affected body site and month of sample collection each were associated with microbiota composition (*P* <0.001). Interestingly, in our data the cutaneous microenvironment (dry vs sebaceous) is not an important factor in accounting for variability of the skin microbiota (*P* >0.05). We further examined the association of the microbiota with the collection date for possible biases. We re-evaluated the model by considering only lesion and unaffected specimens (which are collected from the same individual) and stratifying the analyses by the subject. Under the modified model, the month of collection was not significant, suggesting that the association we originally observed is entirely due to the high intersubject variability, which is captured in part by the collection date variable.

In terms of body sites, the head harbors the most distinct microbiota from the other sites, as expected [19,48], while the lower and upper extremities are highly similar to each other (Table 4). Thus, the interactions of psoriasis status with the recorded clinicodemographic variables were not significantly associated with the cutaneous microbiota distribution.

**Table 4:** ***Post hoc *** **analysis of body site differences**

**Body site comparison**	**Sum of squares**	**Pseudo-F-statistic**	***P *** **value**^**a**^
Body/head	0.481	1.585	**0.018**
Body/upper extremity	0.441	1.465	**0.036**
Head/lower extremity	0.599	1.959	**0.006**
Head/upper extremity	0.586	1.938	**0.006**
Body/lower extremity	0.394	1.298	0.168
Lower extremity/upper extremity	0.340	1.122	0.749

^a^*P* values are based on 1,000 permutations per pairwise comparison and are adjusted for multiple testing using the Bonferroni method. Significant values are in bold.

### Correlation analysis of psoriasis severity

An important prospect of studying the microbiota in psoriatic lesions is to identify taxa that correlate with disease severity, which may lead to better understanding of microbial roles in disease progression. We examined the identified distinct cutaneotypes for association with disease severity, as measured by the PASI, BSA and PGA scores. The cutaneotypes were not significantly associated with any of these severity measures (Additional file 1: Figure S6). However, several specific major taxa present across many taxonomic levels are associated with severity (Table 5). We did not find any taxa that were simultaneously correlated with all three measures of severity. This suggests that there is a weak link between the clinical assessment of disease severity and microbial colonization of the lesions.

**Figure 6:**
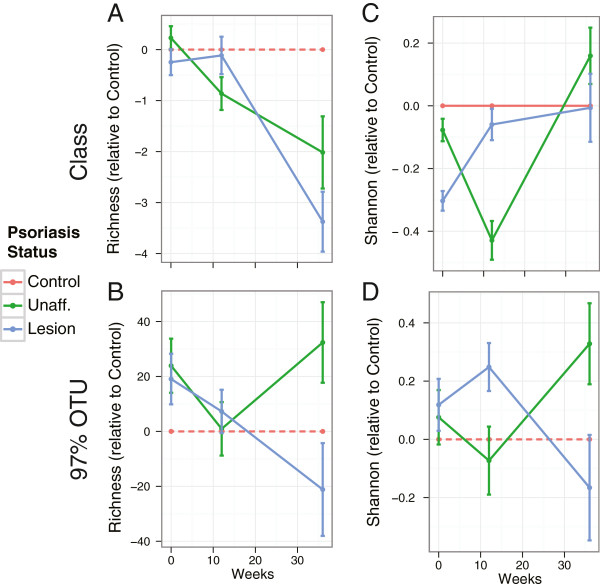
**α Diversity trends in the longitudinal cohort.** For the longitudinal cohort, we show the α diversity (**(A,B)**: richness, **(C,D)** Shannon) of unaffected (green) and lesion (blue) samples relative to the control group at the class level (A,C) and for 97% operational taxonomical units (OTUs) (B,D). At all timepoints, the data show no statistically significant intergroup differences in α diversity.

**Table 5:** Correlation of specific taxa with psoriasis severity

**Group**	**Subgroup**	**PASI**		**BSA**		**PGA**	
		**ρ**	***P *** **value**^**a**^	**ρ**	***P *** **value**^**a**^	**ρ**	***P *** **value**^**a**^
Phylum	Acidobacteria	-0.50	0.003*	-0.54	0.032*	-0.33	0.080
	Proteobacteria	-0.33	0.087	-0.03	0.893	-0.43	0.018*
Class	Acidobacteria_Gp4	-0.40	0.029*	-0.20	0.688	-0.29	0.094
	Sphingobacteria	-0.20	0.370	-0.13	0.688	-0.37	0.039*
	Alphaproteobacteria	-0.25	0.244	-0.07	0.751	-0.40	0.035*
	Betaproteobacteria	-0.45	0.018*	-0.21	0.688	-0.39	0.035*
Order	Gp4	-0.40	0.038*	-0.20	0.623	-0.29	0.102
	Sphingobacteriales	-0.20	0.344	-0.13	0.645	-0.37	0.048*
	Bacillales	-0.01	0.954	-0.29	0.501	0.39	0.048*
	Rhizobiales	-0.26	0.332	-0.11	0.645	-0.43	0.033*
	Sphingomonadales	-0.20	0.344	0.04	0.847	-0.34	0.049*
	Burkholderiales	-0.43	0.036*	-0.29	0.501	-0.36	0.048*
Family	Methylobacteriaceae	-0.22	0.491	-0.12	0.856	-0.42	0.038*
	Comamonadaceae	-0.22	0.491	0.07	0.902	-0.51	0.005*
97% OTU	Methylobacterium;484	-0.22	0.346	-0.16	0.705	-0.38	0.036*
	Gp4;3855	-0.45	0.022*	0.05	0.952	-0.32	0.085
	Schlegelella;13613	-0.04	0.927	0.20	0.705	-0.39	0.036*
	Methylobacterium;24283	-0.15	0.704	-0.08	0.912	-0.40	0.036*

^a^Only taxa that are significant at 5% FDR (*) for at least one measure of severity are shown.

*BSA* body surface area, *OTU* operational taxonomical unit, *PASI* Psoriasis Area and Severity Index, *PGA* physician global assessment.

### Longitudinal analysis of the cutaneous microbiota

The longitudinal basis of sample collection allowed us to assess the effects of anti-inflammatory therapies on the composition of the microbiota communities. The psoriasis patients showed an overall improvement in the clinical severity of the lesions during the course of the treatment (Additional file 1: Table S6). Because we were only able to obtain follow-up samples from 17 and 9 subjects at 12-week and 36-week timepoints, we used the control specimens to reduce variability in the α diversity estimates. Our initial examination of the α diversity in the longitudinal cohort demonstrated high variability in the measurements of richness and Shannon index. Therefore, we looked for a way to minimize the variability in the data by restricting our analysis to triplets and viewing them in the light of natural variability that is represented by the control subjects. To do so, for each triplet we subtracted the α diversity of the control specimen from those of the unaffected and lesion specimens. The utility of this approach is in that in our case it allowed for consistent longitudinal trends to emerge in otherwise highly variable data. Thus, the longitudinal α diversity results are presented in terms of α diversity relative to the controls in each triplet (Figure 6). Although no statistically significant difference was observed between lesion and unaffected groups or longitudinally within groups, we observed several consistent patterns. Richness decreased over time (and treatment) in both lesion and unaffected specimens at all taxonomical levels, except for 97% identity OTUs, where the lesion demonstrated similar decrease, while the unaffected cutaneous flora rebounded to baseline levels at week 36. We also observed an increase in evenness (Shannon index), followed by a later decline. Both observations are consistent with findings in the cross-sectional cohort and lead to the similar conclusion that the abundance of a taxon or a group of taxa was increased, leading to elimination of other taxa (decreasing richness) and lower abundances of the others (decline in evenness). These patterns are preliminary and will require a larger cohort to confirm or reject.

**Figure 7:**
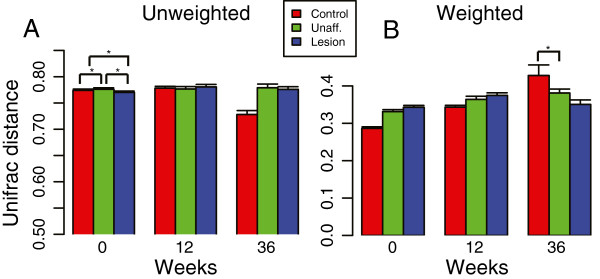
**Intragroup β diversity trends in the longitudinal cohort. (A)** Unweighted, and **(B)** weighted intragroup Unifrac distances. We show β diversity at three timepoints in relation to treatment of the patients with psoriasis, at 0 (pretreatment), 12, and 36 weeks of therapy. An asterisk (*) indicates comparisons that are significant at the 5% α level.

The weighted and unweighted Unifrac intragroup β diversity estimates were similar among the three groups at all sampling times (Figure 7). Significant intragroup differences only were observed at baseline (Figure 7A) in case of unweighted Unifrac. One explanation for the absence of robust statistical differences in α and β diversity between the skin types is insufficient power in the longitudinal study.

**Figure 8:**
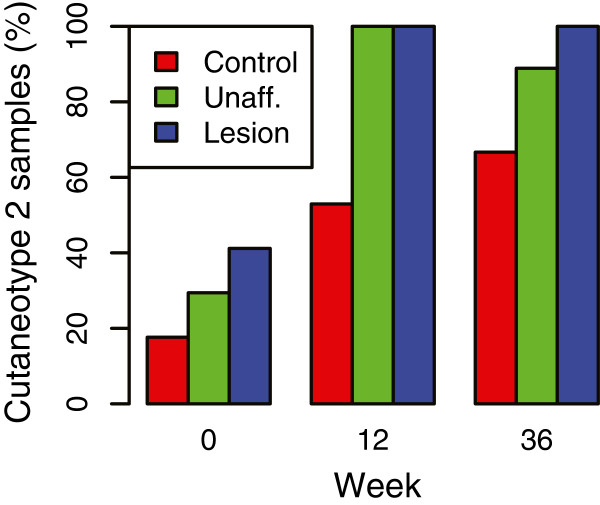
**Distribution of the longitudinal samples in cutaneotype 2.** The cutaneotype prevalence in the longitudinal cohort reflects that of the cross-sectional study (Figure 5C). Cutaneotype 2 is most prevalent in the unaffected and lesion specimens. The frequency of cutaneotype 2 is significantly different (*P* <0.005, Fisher exact test) at week 12 in control vs lesion and in control vs unaffected specimens. Other comparisons are not statistically significant.

However, the longitudinal samples allowed us to confirm the observation that cutaneotype 2 is most prevalent in psoriasis subjects even in the course of treatment. We found that although the prevalence changed over time, the relative trend of increased prevalence of cutaneotype 2 in unaffected and lesion specimens was consistent at all three timepoints (Figure 8). As can be seen from examining just the controls, subjects may switch from cutaneotype 1 to 2 within the course of the experiment. The source for this dynamic behavior is unknown and cannot be determined from the data we have, but suggests that in the skin as with other body sites (for example, the vagina; see [3]), the major clustering types may shift for an individual. This may be an important observation. This serves as an initial evidence of utility of cutaneotype clustering, which needs to be further examined in the future. A possible limitation to this observation is that the apparent increase in the proportion of cutaneotype 2 samples may in part be due to decreasing number of triplets over time in the longitudinal cohort (17 at baseline and 12-week mark and 9 at 36-week mark). A larger longitudinal cohort will be needed to see if this is a real limitation.

**Figure 9:**
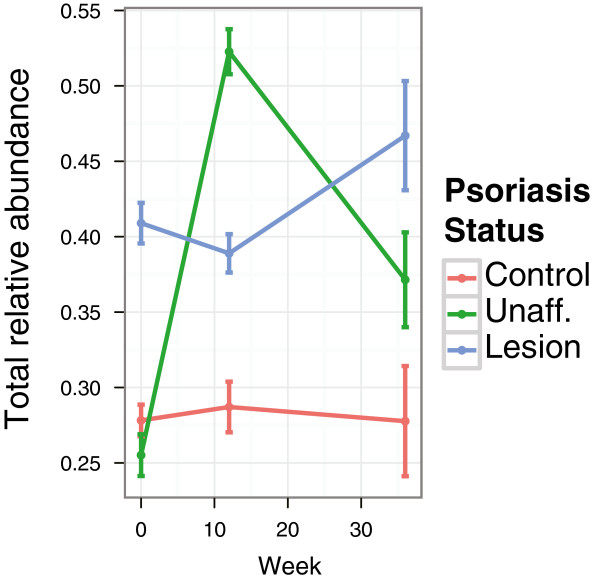
**Relative abundance of skin-associated genera in longitudinal samples.** The combined abundance (± SEM) of four skin-associated genera (*Corynebacterium*, *Propionibacterium*, *Staphylococcus* and *Streptococcus*) is shown over the course of the longitudinal study in three skin types. Control skin demonstrates a stable relative abundance of these bacteria, while variability is evident in unaffected and lesion skin. Lesional microbiota shows a dramatic increase in these bacteria at all timepoints.

We also examined the longitudinal stability of the observed differential abundance of skin-associated genera (*Corynebacterium*, *Propionibacterium*, *Staphylococcus,* and *Streptococcus)*. We found that the total combined relative abundance of these taxa remained surprisingly stable in the control specimens throughout the course of the experiment, while their abundance in the lesion and unaffected samples varied over time and was consistently different from that of control (Figure 9). The lesion specimens contained a higher proportion of bacteria belonging to these skin genera, and that proportion increased slightly from 40.9% at baseline to 46.7% at week 36. The abundance of these bacterial genera in the unaffected skin (25.5%) was similar to the control (27.8%) at baseline, but increased dramatically after 12 weeks of treatment (52.3%) before declining (37.1%) after further 24 weeks of treatment. This suggests that normally stable microbiota are perturbed by the treatment. A larger cohort of subjects is needed to examine the clinical significance, strength, and stability of such perturbation.

## Discussion

Although the taxa represented in control, unaffected, and lesion sites are highly similar, regular patterns were observed, which we captured as cutaneotypes. This definition of cutaneotypes is potentially useful, but must be confirmed in subsequent studies with larger populations and longer follow-up. It is particularly promising that we are able to associate the *Firmicutes* and *Actinobacteria*-rich cutaneotype with psoriasis status, an association that also is maintained in our small-scale longitudinal study.

Further promising evidence of the utility of examining the cutaneous microbiome for markers of disease is provided by identifying differential colonization of the major skin taxa (*Corynebacterium*, *Propionibacterium*, *Staphylococcus,* and *Streptococcus*), not only in the lesions, but also on the unaffected skin. The increase in the combined relative abundance of these genera in psoriasis is complemented by decreases in other genera, such as *Cupriavidus*, *Methylobacterium,* and *Schlegelella*, which all carry a strong diagnostic signal pertinent to the disease. The absence of two specific OTUs (*Gp4* and *Schlegellela*) likewise provides an independent strong binary diagnostic signal. These two taxa, which correlated best with psoriasis, *Gp4* and *Schlegelella*, were either not found in the HMP subjects [4] (*Gp4*), or found only rarely (in 3 of 664 samples). Although they may be transients or contaminants, they may also serve as markers of decreased diversity associated with abnormal conditions in the skin of psoriasis patients and specifically at the lesion sites. The four-genera abundance-based biomarker appears to capture similar information afforded by the discovered cutaneotypes, while the double-positive binary predictor is not driven by the cutaneotype structure. Although associations of specific taxa with disease severity were not robust, we predict that cutaneous microbiota composition will provide additional insight to understand mechanistic aspects of the continued cutaneous immune response.

The HMP study of healthy individuals yielded only low levels of cutaneous *Proteobacteria*[19], differing from both our own clinical observations and other studies in psoriasis [2]. Cutaneotype 1, most prevalent in our healthy control subjects, tends to have high *Proteobacteria* abundance. That overall composition in our controls differed from the healthy persons in the main HMP cohort could reflect differences in geography, climate, study subject characteristics, sampling sites and techniques, as well as sequencing and analytical methodologies. However, the sampling, sequencing, and analytic pipelines were nearly identical to the HMP protocol.

The longitudinal studies also indicate that the psoriasis treatments reduce richness and increase evenness, at least transiently, in both the lesion and the unaffected cutaneous communities. Since the treatments are systemic and not local, such a generalized response was expected; nevertheless, its presence further validates our initial findings. The extreme dynamism of the major genera in the clinically unaffected skin with early treatment *vis a vis* the lesion sites indicates substantial instability or transition state at such sites.

Prior studies of the cutaneous microbiota have indicated extensive interindividual variability [2,6-8,11-16], especially in relation to other body sites, as shown by the HMP data [19]. The comparison of lesional and unaffected specimens from the same subject in our study mitigates this issue. The necessary comparisons between diseased and control subjects are affected by the interindividual variation; inclusion of 51 pairs helps control for this, but even larger study sizes would be better. That the clinically unaffected samples are interposed between control and lesion in our analyses, provide some confidence in the biologic plausibility of our approach and interpretations.

## Conclusions

In this work, we present the first comprehensive analysis of the community structure of the cutaneous microbiota in psoriasis patients. Although we analyzed the data for 51 triplets of control, unaffected, and lesion specimens, the inherent heterogeneity of the skin microbiota [19] as well as the heterogeneity of the disease [1,2,5] requires still-larger datasets to strengthen our conclusions. Nonetheless, our robust triplet study design and rigorous exclusion criteria that preclude subjects with recent relevant or antibiotic treatment, allowed us to perform a preliminary examination of the changes in the microbial ecology of cutaneous sites in response to psoriasis.

Consistent decreases of taxonomic and species (OTU) level diversity in terms of both evenness and richness provide evidence that psoriasis is a stress condition that selects against the normally present cutaneous bacterial diversity. Importantly, the effect is observed not only at the affected sites (lesion), but also at the clinically unaffected contralateral skin site (unaffected), albeit to a lesser degree. These observations indicate that psoriasis is a condition that affects the composition of the microbiota as a whole, leading to shifts of the clinically unaffected microbiota toward that of the lesions, and not specifically limited to the lesion sites.

The skin sites showed a progressive increase in intragroup diversity from control to unaffected to lesion. This observed increase in specimen heterogeneity obtained from affected individuals provides further evidence for ecosystem disruption in the clinically unaffected sites, and indicates the multiple cutaneous responses to the selectional stress introduced by the psoriatic immunopathophysiology [49-53]. Diseased tissue selects for different microbiota than healthy, resulting from altered physical, clinical, and immunological properties. The differential compositions that we observed are *a priori* evidence for the power of disease-specific selection. Another plausible but less likely or exciting alternative is that the lesion sites serve as the reservoirs for transfer of the microbes to the unaffected skin sites by scratching, touching, washing or clothes, which result in apparent decrease in diversity at these sites. The design of our study does not provide for a means for distinguishing between these two hypotheses.

Despite many limitations inherent to such observational studies, our findings advance understanding of the effects of psoriasis on the compositional status of the cutaneous microbiota. We find substantial impact, which if confirmed, may have important diagnostic, preventive, and potentially therapeutic implications. Future studies might also include metagenomic and metatranscriptomic analyses if limitations in DNA quantity and quality from cutaneous samples do not become a significant impediment.

## Availability of supporting data

Clinicodemographic information on the subjects of this study and sequences for this study are deposited for controlled public access through dbGap accession phs000251.

## Abbreviations

ANOVA: Analysis of variance; AUC: Area under curve; BSA: Body surface area; dbGaP: Database of genotypes and phenotypes; FDR: False discovery rate; gDNA: Genomic DNA; HMP: Human microbiome project; HSD: Honestly significant difference; IRB: Institutional review board; JCVI J: Craig Venter Institute; JTC: Joint technology center; MANOVA: Multivariate ANOVA; NIH: National institutes of health; OTU: Operational taxonomic unit; PAM: Partitioning around medoids; PASI: Psoriasis area and severity index; PC: Principal component; PCoA: Principal coordinates analysis; PCR: Polymerase chain reaction; PGA: Physician global assessment; PSA: Prostate specific antigen; QIIME: Quantitative insights into microbial ecology; ROC: Receiver operating characteristic; rRNA: Ribosomal RNA.

## Competing interests

The authors declare that they have no competing interests.

## Authors’ contributions

AVA designed the downstream informatics and statistical analyses, interpreted the results and wrote the manuscript. GIP-P and AD’S managed clinical data, BS oversaw subject recruitment and diagnosis, ZG managed clinical samples, MB, KL, and BAM performed the sequencing assays and upstream informatics analysis, MJB and GIP-P designed the clinical study, interpreted the results, MJB, GIP-P, BAM and SB helped to draft the manuscript. All authors read and approved the final manuscript.

## Supplementary Material

Additional file 1 PDF Supplemental Figures, Tables and Methods. Click here for file
