# Supplementary material for: Community differentiation of the cutaneous microbiota in psoriasis
Source: Microbiome. 2013 Dec 23;1:31. doi: 10.1186/2049-2618-1-31 (PMC4177411; doi:10.1186/2049-2618-1-31)
Supplement: Additional file 1 — PDF Supplemental Figures, Tables and Methods. [file 2049-2618-1-31-S1.pdf]

Supplementary Figures

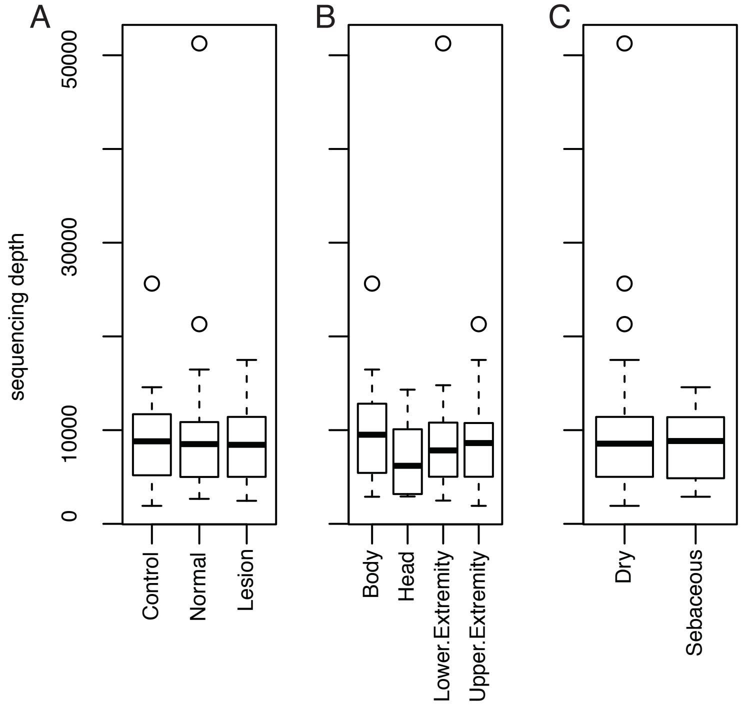

Supplemental Figure 1. Sequencing effort depth. The box-and-whiskers plots show the distribution of sequencing depth across clinical skin type (**Panel A**), body site (**Panel B**), and skin micro-environment(**Panel C**).

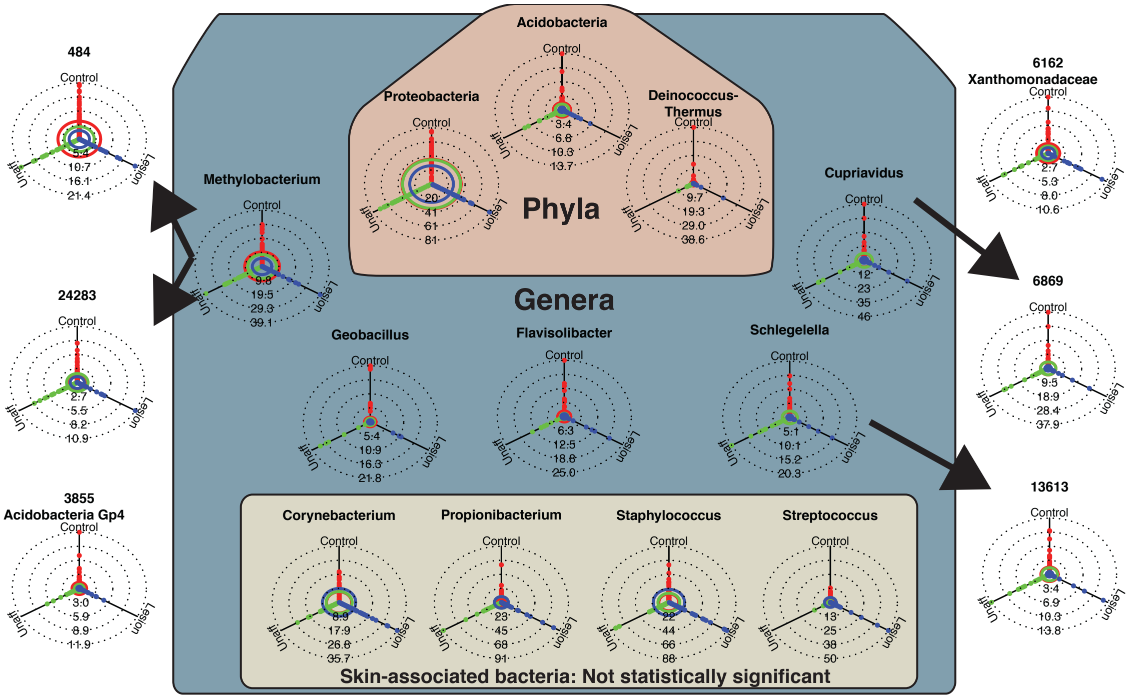

Supplemental Figure 2. Target plots. The target plots show relative abundance as a fraction of each taxon in each sample (points). Samples are stratified by color and dimension according to tissue status (Lesion – blue, Unaffected – green, Control – red). Circles of the corresponding colors show the means across all samples in the tissue status groups. The major cutaneous genera: *Corynebacterium*, *Propionibacterium*, *Staphylococcus*, and *Streptococcus* were not significantly different between the groups. All of the other taxa shown are significantly different according to Kruskal-Wallis ANOVA at a 5% false discovery rate.

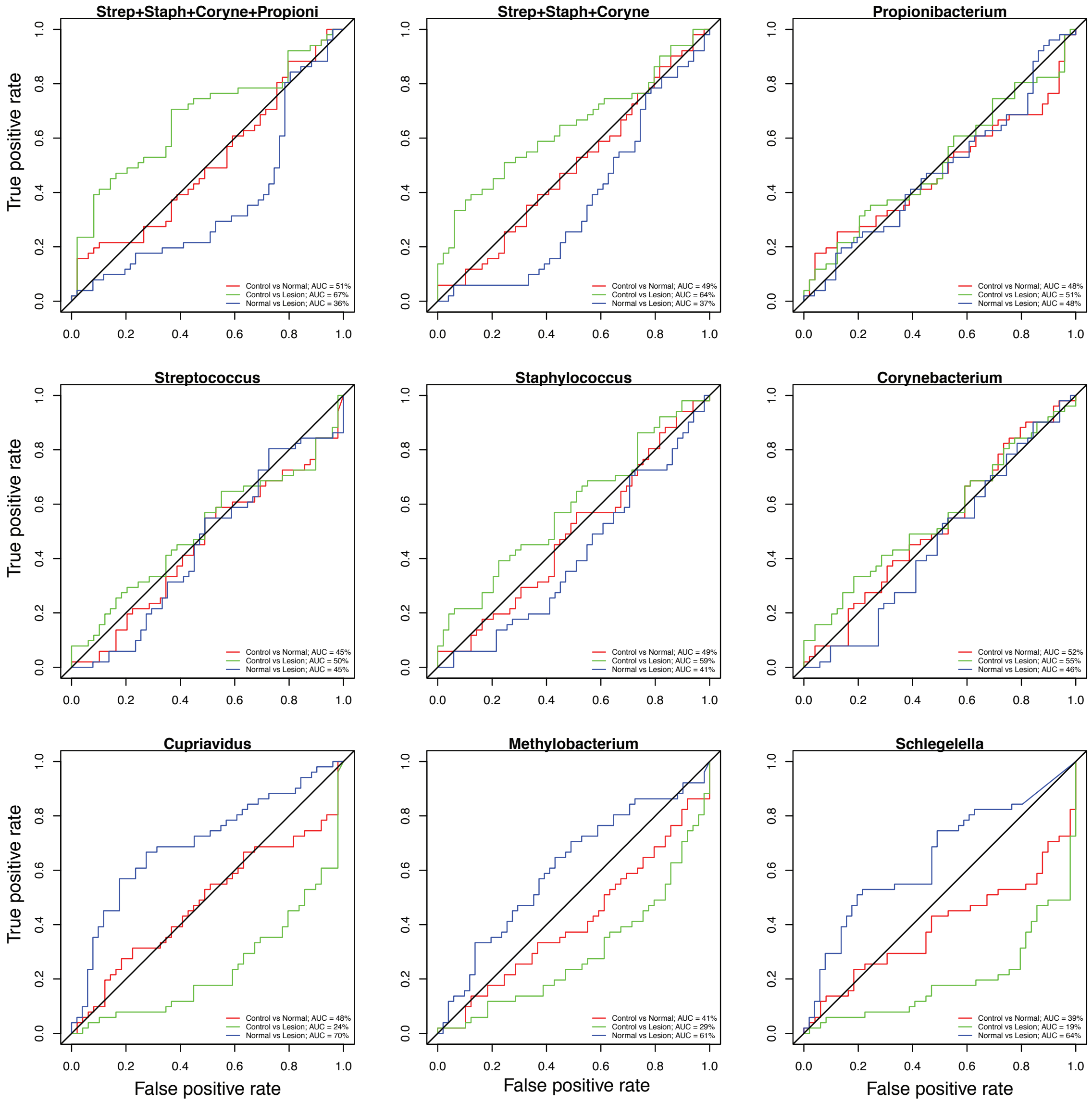

Supplemental Figure 3. Univariate classification signal of selected genera. We used the abundance of several candidate genera as univariate predictors of the psoriasis status for 3 predictive tasks Control vs. Unaffected, Control vs. Lesion and Unaffected vs. Lesion. Area under receiver-operator characteristic curve (AUC) as shown on the plot for each predictor was computed by considering all possible relative abundance cut-off and computing the true-/false-positive rates. The AUCs is a measurement of classification signal carried by the corresponding predictor, such that the strongest predictor has AUC

100% and a random predictor has AUC 50%. The AUCs for the three predictive tasks are reported on each panel. The major skin-associated taxa (*Streptococcus*, *Staphylococcus*, *Corynebacterium*, *Propionibacterium*) do not significantly classify the specimens in univariate abundance-based classifiers (AUC close to that obtainable by chance is 50%). In contrast, the genera *Cupriavidus*, *Methylobacterium*, and *Schlegelella* better classify the mix of specimens. Combined (by direct sum of relative abundances) classification signals of the skin-associated taxa achieves a similar classification strength. A further exploration of the combined classifier reveals that *Propionibacterium* can be dropped without loss of signal.

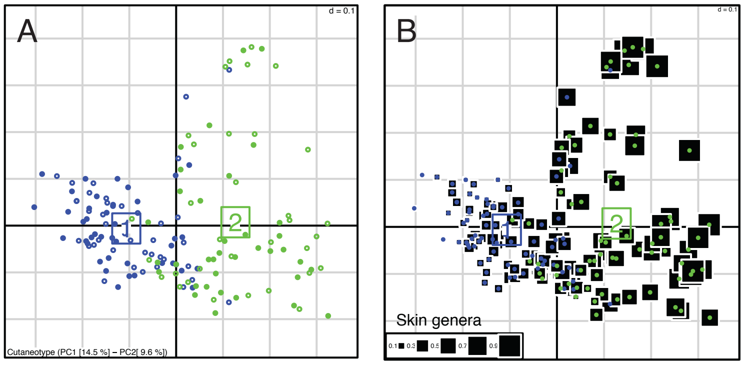

Supplemental Figure 4. Representation of cutaneotypes on PCoA projection. Based on examination of the eigen-value plot we determined that a significant drop in variability explained occurs after inclusion of just 2 principal axes. **Panel A:** Representation of the cutaneotypes (1 – green, 2 – blue) on the first two axes of the PCoA. The cutaneotype 1 samples tend to the left side of the plane, while cutaneotype 2 samples are on the left. **Panel B:** Combined relative abundance of the skin-associated genera (*Streptococcus*, *Staphylococcus*, *Corynebacterium*, *Propionibacterium*) is represented by the size of the squares. The coordinated plot of cutaneotypes and relative abundance of skin-associated genera, suggests that the clustering may be related to change in abundance of these genera.

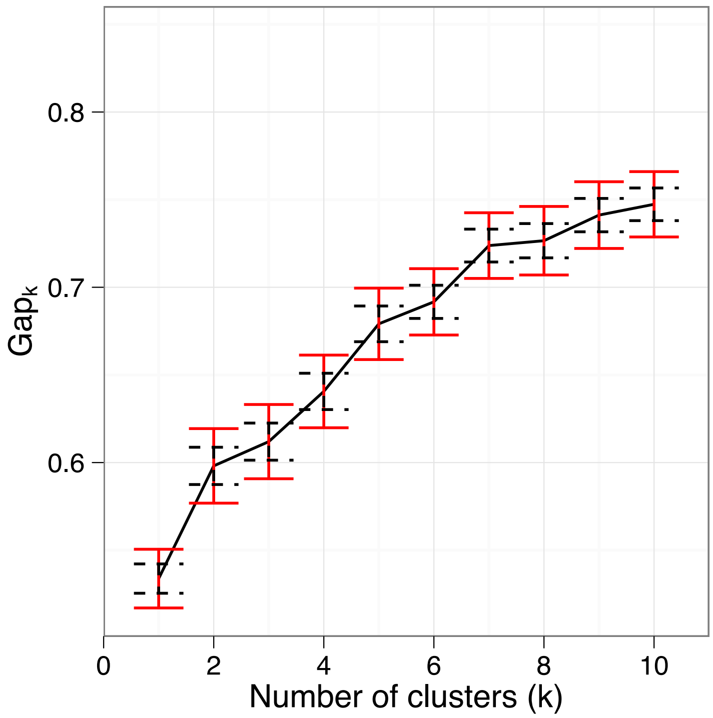

Supplemental Figure 5. Gap statistic for cutaneotype clustering. See Supplemental Methods section "Validation of clustering using the gap statistic " for methodology details. The points on the curve represent the estimates of gap statistic for a given number of clusters. The standard error intervals are shown as dotted bars (1 – S.E.) and red bars (2 - S.E.). Based on the 2-standard error interval heuristic, we observe that with 2 clusters the gap statistic is significantly different from gap statistic with no clustering (i.e. 1 cluster) and overlaps the interval for the gap statistic for 3 clusters. Hence we conclude that 2 is the adequate number of clusters to represent our data.

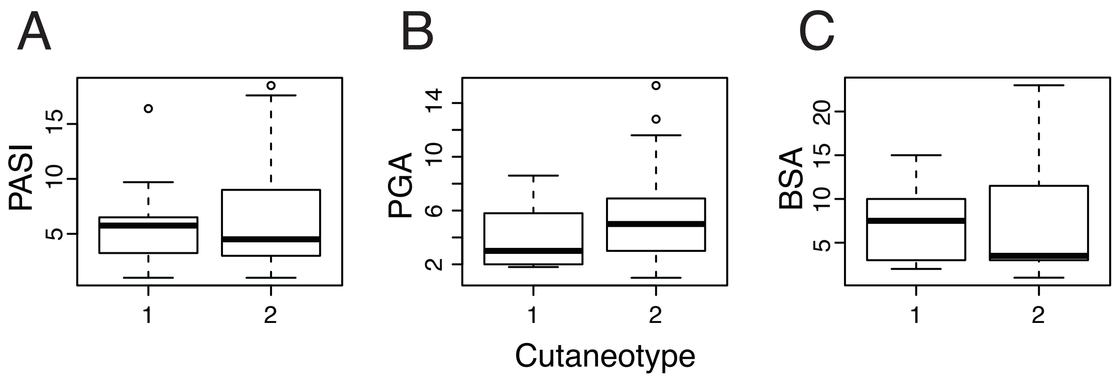

Supplemental Figure 6. Association of cutaneotypes with psoriasis severity. The box-and-whiskers plots show the distribution of severity scores across cutaneotypes, as measured by PASI (**Panel A**), PGA (**Panel B**), or BSA (**Panel C**). There was no significant association between cutaneotype and psoriasis severity.

Supplementary Tables

Supplemental Table 1. Matching of psoriasis lesions to control sites and skin environment

	Subject identifier		Actual site		Standard Site	Skin environment	
Triplet	Control	Psoriasis	Control	Psoriasis		Control	Psoriasis
1	10	62	scalp	scalp	Head	Sebaceous	Sebaceous
2	3	46	elbow	elbow	Upper.Extremity	Dry	Dry
3	14	63	elbow	elbow	Upper.Extremity	Dry	Dry
5	11	69	knee	shin	Lower.Extremity	Dry	Dry
6	7	56	abdomen	back	Body	Dry	Sebaceous
7	31	77	elbow	elbow	Upper.Extremity	Dry	Dry
8	8	74	knee	knee	Lower.Extremity	Dry	Dry
9	34	64	knee	knee	Lower.Extremity	Dry	Dry
10	22	71	knee	foot	Lower.Extremity	Dry	Dry
11	35	68	elbow	elbow	Upper.Extremity	Dry	Dry
13	21	57	scalp	scalp	Head	Sebaceous	Sebaceous
14	34	67	elbow	forearm	Upper.Extremity	Dry	Dry
15	8	76	elbow	elbow	Upper.Extremity	Dry	Dry
16	1	60	knee	knee	Lower.Extremity	Dry	Dry
17	3	48	knee	knee	Lower.Extremity	Dry	Dry
18	33	44	elbow	elbow	Upper.Extremity	Dry	Dry
20	16	47	elbow	elbow	Upper.Extremity	Dry	Dry
21	14	75	knee	knee	Lower.Extremity	Dry	Dry
22	15	43	elbow	elbow	Upper.Extremity	Dry	Dry
23	32	45	forearm	forearm	Upper.Extremity	Dry	Dry
24	10	66	elbow	hand	Upper.Extremity	Dry	Dry
25	20	73	elbow	forearm	Upper.Extremity	Dry	Dry
26	2	65	scalp	face	Head	Sebaceous	Sebaceous
27	16	58	abdomen	back	Body	Dry	Sebaceous
28	12	70	elbow	shoulder	Upper.Extremity	Dry	Dry
29	15	49	knee	knee	Lower.Extremity	Dry	Dry
30	12	54	knee	thigh	Lower.Extremity	Dry	Dry
31	29	50	elbow	elbow	Upper.Extremity	Dry	Dry
32	25	52	abdomen	abdomen	Body	Dry	Dry
33	34	53	back	back	Body	Sebaceous	Sebaceous
34	34	59	back	back	Body	Sebaceous	Sebaceous
35	22	78	elbow	elbow	Upper.Extremity	Dry	Dry
36	24	79	abdomen	back	Body	Dry	Sebaceous
37	40	80	knee	leg	Lower.Extremity	Dry	Dry
38	6	81	abdomen	back	Body	Dry	Sebaceous
39	19	82	knee	leg	Lower.Extremity	Dry	Dry
40	35	55	knee	leg	Lower.Extremity	Dry	Dry

41	27	61	abdomen	back	Body	Dry	Sebaceous
42	4	51	knee	knee	Lower.Extremity	Dry	Dry
43	41	72	knee	shin	Lower.Extremity	Dry	Dry
44	36	83	elbow	forearm	Upper.Extremity	Dry	Dry
45	23	84	knee	leg	Lower.Extremity	Dry	Dry
46		85		arm	Upper.Extremity	NA	Dry
47	42	86	elbow	elbow	Upper.Extremity	Dry	Dry
48	13	87	elbow	elbow	Upper.Extremity	Dry	Dry
49	24	88	elbow	elbow	Upper.Extremity	Dry	Dry
50	33	89	abdomen	back	Body	Dry	Sebaceous
51	38	90	knee	leg	Lower.Extremity	Dry	Dry
52	30	91	knee	leg	Lower.Extremity	Dry	Dry
53	19	92	abdomen	abdomen	Body	Dry	Dry
54		93		thigh	Lower.Extremity	NA	Dry

Supplemental Table 2. The samples included in longitudinal samples are shown.

Triplet	Baseline	12 weeks	36 weeks	Control	Psoriasis	Control	Psoriasis	Standard Site	Control	Psoriasis
1	x	x	x	14	44	elbow	elbow	Upper.Extremity	Dry	Dry
2	x	x	x	28	45	elbow	forearm	Upper.Extremity	Dry	Dry
3	x	x	x	3	46	elbow	elbow	Upper.Extremity	Dry	Dry
4	x	x	x	9	48	knee	knee	Lower.Extremity	Dry	Dry
5	x	x	x	9	50	elbow	elbow	Upper.Extremity	Dry	Dry
6	x	x	x	5	51	knee	knee	Lower.Extremity	Dry	Dry
7	x	x	x	27	53	abdomen	back	Body	Dry	Sebaceous
8	x	x	x	6	78	elbow	elbow	Upper.Extremity	Dry	Dry
9	x	x	x	6	81	abdomen	back	Body	Dry	Sebaceous
10	x	x		16	47	elbow	elbow	Upper.Extremity	Dry	Dry
11	x	x		12	54	knee	thigh	Lower.Extremity	Dry	Dry
12	x	x		24	79	abdomen	back	Body	Dry	Sebaceous
13	x	x		17	82	knee	leg	Lower.Extremity	Dry	Dry
14	x	x		26	83	elbow	forearm	Upper.Extremity	Dry	Dry
15	x	x		18	85	elbow	arm	Upper.Extremity	Dry	Dry

16	x	x	39	86	elbow	elbow	Upper.Extremity	Dry	Dry
17	x	x	37	91	knee	leg	Lower.Extremity	Dry	Dry

Supplemental Table 3. Characteristics of the cross-sectional cohort

	Patients (n=51)	Controls (n=35)
**Age Mean (years) ± SD**	49.1 ± 16.4	46.1 ± 16.0
**Ethnicity (%)**		
Caucasian	74.5	71.4
African American	7.8	8.6
Hispanic	13.7	17.1
Asian	3.9	2.9
**Gender (%)**		
Male	76.5	74.3
Female	23.5	25.7
**Family History of Psoriasis (%)**	47.1	5.7

Supplemental Table 4. Number of sequences sampled for significant OTUs per skin type

	Control	Unaffected	Lesion
**Gp4 (OTU 3855)**	**5,934**	**4,211**	**2,205**
**Schlegelella (OTU 13613)**	**6,456**	**7,916**	**2,803**

Supplemental Table 5. Representative sequences of the OTUs forming the double-positive diagnostic predictor.

Taxon	Amplified representative sequence
**Gp4 (OTU 3855)**	GAATCAACGCTGGCGGCGTGCCTCAGACATGCAAGTCGAACGATTA AGACTTCCTTCGGGAAGTGTATAAAGTGGCGCACGGGTGAGTAACA CGTAAGTAATCTACCCTCGAGTGGGGAATAACATCGGGAAACCGAT

	GCTAATACCGCATAACGCAGCGGCACCGCAAGGTGACAGTTGTTAA<br>AGGAGCAATCCGCTTGAGGAGGAGCTTGCGGCAGATTAGCTAGTTG<br>GTGAGGTAATGGCTCACCAAGGCGACGATCTGTATCCGGCCTGAGA<br>GGGCGGTCGGACACACTGACACTGAATAACGGGTCAGACTCCTACG<br>GGAGGCAGCAGTCGGGAATTTTGGGCAATGGGCGAAAGCCTGACCC<br>AGCAACGCCGCGTGAGGGATGAAGTATTTCGGTATGTAAACCTCGA<br>AAGAATGGGAAGAATAAATGACGGTACCATTTATAAGCTCCGGCTA<br>ACTACGTG
**Schlegelella (OTU 13613)**	GATTGAACGCTGGCGGCATGCTTTACACATGCAAGTCGAACGGCAG<br>CGCGGGCTTCGGCCTGGCGGCGAGTGGCGAACGGGTGAGTAATGCA<br>TCGGAACGTGCCCAGTAGTGGGGGATAGCCCGGCGAAAGCCGGATT<br>AATACCGCATACGACCTGAGGGTGAAAGCGGGGGACCGAAAGGCCT<br>CGCGCTATTGGAGCGGCCGATGTCGGATTAGCTAGTTGGTGGGGTA<br>AAGGCTTACCAAGGCGACGATCCGTAGCTGGTCTGAGAGGACGACC<br>AGCCACACTGGGACTGAGACACGGCCCAGACTCCTACGGGAGGCAG<br>CAGTGGGGAATTTTGGACAATGGGGGCAACCCTGATCCAGCCATGC<br>CGCGTGCGGGAAGAAGGCCTTCGGGTTGTAAACCGCTTTTGTTCGG<br>GAAGAAATCCTCTGGGCTAATACCCCGGGGGGATGACGGTACCGGA<br>AGAATAAGCACCGGCTAACTACGTG

Supplemental Table 6. Average PASI score for psoriasis subjects in the longitudinal study.

	Baseline	12 weeks	36 weeks
Mean PASI	11.5	2.9	3.1
S.E.M.	2.7	0.8	1.9
N	17	17	9

Supplemental Methods

Validation of clustering using the gap statistic

The gap statistic [1] is an alternative to the Calinski-Harabasz index [2] approach to establish the presence of clustering and of adequate number of clusters. Rather than examining the ratio of within to between cluster dispersion, the gap statistic compares only the within distances with that expected by random chance. Another difference in the two approaches lies in the exact way in which the optimum is determined. In case of Calinski-Harabasz index, the minimum is used. The criteria for gap statistic are numerous. Tibshirani et al. propose to the use one-standard deviation rule, i.e. to call

optimum number the smallest k such that the gap statistic for k falls inside the one standard error interval around the gap statistic for k+1. However, the authors also note that the choice to employ one standard error unit is arbitrary and can be adjusted to particular applications. We computed the gap statistic to define the number of cutaneotypes present in our data (**Supplemental Figure 4**). Using the one standard deviation heuristic seven cutaneotypes are appropriate. Since this is a very large number, especially in relation to our sample size (151), we choose to employ a more stringent criterion. In a similar way to the original approach, we choose to use 2 standard errors to determine the optimum number of clusters. This approach results in two cutaneotypes as the appropriate number of clusters to use, which is consistent with the result, obtained using the maximum of the Calinski-Harabasz index.

Additional References

1. Tibshirani R, Walther G, Hastie T: **Estimating the number of clusters in a data set via the gap statistic.** *Journal of the Royal Statistical Society: Series B (Statistical Methodology)* 2001, **63:**411-423.
2. Calinski R, Harabasz J: **A dendrite method for cluster analysis.** *Communications in Statistics* 1974, **3:**1-27.
